# Perceptions of Persons With Type 2 Diabetes Treated in Swedish Primary Health Care: Qualitative Study on Using eHealth Services for Self-Management Support

**DOI:** 10.2196/diabetes.9059

**Published:** 2018-03-12

**Authors:** Ulrika Öberg, Ulf Isaksson, Lena Jutterström, Carl Johan Orre, Åsa Hörnsten

**Affiliations:** 1 Department of Nursing Umeå University Umeå Sweden; 2 Arctic Research Centre Umeå University Umeå Sweden; 3 Department of Computer Science and Media Technology Malmö University Malmö Sweden

**Keywords:** eHealth, internet, type 2 diabetes, self-management, primary health care, qualitative research

## Abstract

**Background:**

Digital health services are increasing rapidly worldwide. Strategies to involve patients in self-monitoring of type 2 diabetes (T2D) on a daily basis is of crucial importance, and there is a need to optimize the delivery of care such as self-management support. Digitalized solutions have the potential to modify and personalize the way in which people use primary health services, both by increasing access to information and providing other forms of support at a distance. It is a challenge to integrate core values of person-centered care into digitalized health care services.

**Objective:**

The objective of this study was to describe perceptions of using electronic health (eHealth) services and related technologies for self-management support among people with T2D treated in Swedish primary health care.

**Methods:**

This is a qualitative study based on interviews analyzed using qualitative content analysis conducted among people diagnosed with T2D.

**Results:**

Findings suggest that the participants had mixed feelings regarding the use of digital health services for self-management support. They experienced potentials such as increased involvement, empowerment, and security, as well as concerns such as ambivalence and uncertainty.

**Conclusions:**

Digital health services for self-management are easily accessible and have the potential to reach a wide population. However, targeted training to increase digital skills is required, and personalized devices must be adapted and become more person-centered to improve patients’ involvement in their own care.

## Introduction

Information and communication technology (ICT) for health promotion, disease prevention, and disease management used in health care (electronic health, eHealth) is suggested to have a great potential to improve access, quality, safety and efficiency of care, and further prevention, diagnostics, treatment, and self-management among people with chronic illnesses such as type 2 diabetes (T2D) [1-3]. Until about a decade ago, the idea of allowing a digital device to play a decisive role in how T2D is controlled and monitored was unthinkable. Today, it is booming in health care with a rapid growth and supply of various applications and interactive systems aimed at improving people’s health behavior and supporting self-management in chronic illness [4].

People with T2D and their perceptions of using digital health services and related technology is the objective of this study. Digital health services or eHealth are terms that are used interchangeably in this paper. In these terms, we include using the internet for medical and health information and self-management support via, for example, diabetes websites, using patient portals, blogs, chat rooms, and forums. Furthermore, telehealth, telemedicine, telemonitoring, mobile Health (mHealth), apps, electronic health records, and other uses of digitization could be involved. These technologies are important since they are supposed to provide, improve, and support self-management and the delivery of care at a distance.

Even if developments and implementations of ICT in health care proceed quickly, opinions about the efficiency of eHealth vary among both patients and health care professionals [5-7]. This is a challenge as the use of innovative technologies in health care is not possible without the acceptance of patients and health care professionals. To support people with chronic illness to more readily accept digital health services and to gain the ability and knowledge to use ICT, we need to learn more from these groups of users [8,9]. In this paper, the focus is on people with T2D. One reason is that the prevalence of T2D is increasing with considerable morbidity and mortality, generating a heavy burden both at a personal level and at the health care system in both developed and developing countries [10,11]. In Sweden, it is estimated that 4% to 6% of the population has T2D with mean age for diagnosis of about 63 years. [12].

Self-management is a basic and integrated part of the treatment in T2D. Since it is a progressive disease, it must be complemented with oral antidiabetic agents or insulin injections over time, which could add to the burden of the disease [13-15]. To control the disease progression, people with T2D visit physicians and specialist nurses several times per year to take various tests, adjust medication, and get self-management support aimed at postponing severe complications [16]. People with T2D commonly struggle with complex self-management activities, including healthy eating, physical activity, blood sugar testing, self-monitoring, and medications [17,18]. Therefore, to manage diabetes efficiently on a daily basis over time, person-centered and tailored education and support, as well as collaboration or partnership between patients and health care professionals is recommended [13,19,20].

The various technologies used in digital health services such as the internet, mobile apps, and other kinds of interactive digital tools and devices in health care have a potential to facilitate self-management, which in turn may prevent or postpone disease complications in a chronic disease [21-25]. From an economic perspective, eHealth may lead to better cost-efficiency in the health sector [26], and it has a potential to complement or even substitute several personal contacts with health care professionals [27].

Implementation of ICT is recommended in Swedish health care. The government’s vision is clear—Sweden is to be the best in the world in eHealth by 2025, and this has to be realized by using the potential of digitization and eHealth to help people achieve good and equal health and well-being, as well as develop and strengthen their own resources for increased independence and participation in society [28]. Furthermore, the use of eHealth technology is recommended for both professionals and patients, but also that the care should be person-centered [28-30]. A challenge though is to integrate goals of person-centered care (PCC) in the implementation of digitized self-management support [5]. One core value in PCC is the development of a mutual and respectful partnership between patients and health care professionals. Another is that care plans should be based on patients’ narratives, where a comprehensive view of the patients and autonomy is of great importance [31]. How these core values could be integrated into eHealth-based self-management support in practice is not clearly expressed in policy documents.

Preferences for use of eHealth devices for health information are higher among younger people, while persons 70 years and older, are reported to prefer nondigital modalities for health information even if they are internet users [32]. Furthermore, among adult internet users, differences are reported where women are reported to use eHealth devices more frequently than men. There are also differences based on socioeconomic status (SOS) in favor for those with higher SOS, but no differences based on ethnicity [33]. In T2D, a main barrier has been reported to be lack of access to the internet and poor user-friendliness of Web applications. People with T2D in need of care are reported to be more engaged in long-term use of eHealth devices such as Web applications [34]. People with different diseases may also express different needs and expectations toward self-management and eHealth for self-management purposes. In a study by Huygens et al [35], participants reported that eHealth should not replace but complement personal care. They also reported feelings of anxiety and uncertainty about follow-up of deviant measurements. From Sweden, we have not found any studies regarding perceptions or expectations on use of eHealth devices for self-management support in T2D. The objective of this study was, therefore, to describe perceptions of using eHealth services and related technologies for self-management support among people with T2D treated in Swedish primary health care.

## Methods

### Overview

This study is part of a larger randomized intervention project aimed at designing and implementing person-centered interactive self-management support (iSMS) in primary health care in northern Sweden. The overall project has a cocreation design, and participants’ perceptions are therefore of great value for designing a forthcoming intervention that is registered at ClinicalTrials.gov (NCT03165084).

### Participants and Setting

The participants were treated in primary health care in a county in northern Sweden. Inclusion criteria in this study were Swedish-speaking individuals diagnosed with T2D. In total, 11 people (3 women, 8 men) aged from 50 to 78 years (median 65 years) were interviewed.

The purpose was to reach a purposeful sample with an even gender distribution, but it was difficult to recruit women in the study. The duration of T2D among the participants varied from 4 months up to about 10 years. Of these 11 participants, 7 participants lived together with a partner, while 4 were single. Each participant owned a smartphone. Initially, the aim of the study was presented by the first author at an information meeting held at the Local Diabetes Association, where 4 participants declared their interest in participating. A snowball selection was then used to include the remaining 7 participants into the study, that is, enrolled participants suggested names of other people who could be contacted for interviews.

### Data Collection

The first author conducted interviews with the participants individually, either in their homes (n=8) or at the university (n=3) during 2016. All participants were contacted in person or by telephone in advance. They received information about the study, and date and place for the interview were decided. At the interview session, each interviewee was informed again and had the opportunity to ask questions or withdraw participation. The interviews performed by the first author lasted between 40 and 80 (median=60) min and were digitally recorded. During the interview, a semistructured interview guide was used, as well as an ambition to get answers that were narrative in nature. The opening question was, “If I say information technology and eHealth, what do you think of?” Examples of other questions were as follows:

- “Can you please tell me about your experiences of using digital health services in contacts with care?”
- “Have you ever used any digital technology device in your diabetes self-management? Please, tell me about those experiences.”

Probing questions and prompts were used to deepen the topics and to get answers on issues not already mentioned.

### Data Analysis

The interview data were transcribed verbatim by the first author and analyzed using qualitative content analysis as described by Graneheim and Lundman [36]. Qualitative content analysis is a systematic way to describe variations of content in a verbal or written communication [36,37]. The epistemological basis of qualitative content analysis is that data and interpretation are cocreations between the interviewee and the interviewer, and interpretation during the analysis phase is a cocreation of the researchers and the text [38,39]. The analysis was performed in several steps. First, all text was read through thoroughly to get a sense of the whole. This reading revealed 2 overarching domains—*Potentials* and *Concerns*—into which the text was sorted. The text in each domain was then divided into meaning units consisting of words or sentences related to each other through their content and context.

The identified meaning units were then condensed, that is, made shorter without losing the core meaning, and interpreted and labeled with codes. The codes were sorted, based on similarities and dissimilarities, into 12 subcategories within the 2 domains. The subcategories were then abstracted to 5 categories as follows:

- Potentials
  - Involvement
    - Independence
    - Responsibility
  - Empowerment
    - Knowledge
    - Participation
    - Engagement
    - Freedom
  - Security
    - Confidentiality
    - Privacy
- Concerns
  - Ambivalence
    - Insufficient support
    - Lack of digital skills
  - Uncertainty
    - Distrust of information
    - Unreliability

Following the steps of the analysis should not be seen as a linear process, rather a process of going back and forth between the steps and between original data and analyzed data. All authors also discussed the interpretations within every step of the analysis until consensus was achieved [36].

### Ethical Considerations

The Regional Ethical Review Board at Umeå University approved the study (Dnr 2014-179-31M) and was conducted according to the ethical principles described in the Helsinki Declaration [40]. Before giving informed consent, the participants received oral and written information. It was emphasized that participation was voluntary and that they could withdraw from the study at any time without giving explanation; they were also assured of confidentiality. The transcripts were made anonymous by removing personal information. In addition, quotations were made anonymous with small changes in wordings that did not alter their core meaning.

## Results

A total of 5 categories within the domains *Potentials* and *Concerns* were identified in the analysis. The results were divided into 2 domains, 5 categories and 12 subcategories. Each subcategory is further enlightened by quotations from the original interviews in the following text.

### Potentials

Within the domain *Potentials*, which referred to the positive perceptions of using digital health services as self-management support, the categories *Involvement*, *Empowerment*, and *Security* were highlighted.

#### Involvement

The importance of being involved in decisions about medication and in discussions about self-management and goals—for example, blood sugar levels—were highlighted. Some had negative perceptions from previous health care contacts when health care professionals made decisions “over their heads.” The subcategories related to this category are *Independence* and *Responsibility*.

##### Independence

*Independence* included striving to handle all demands related to the disease and was expressed as being natural. However, sometimes, social demands made it difficult to remember or prioritize self-management. Using digital health services was described as a key to try harder and as something positive. Some were willing to pay for digital and technological tools that could provide insights and motivation to self-manage their chronic condition:

I use and have paid for an app on my smartphone, so I can monitor my weight, daily steps and of course my blood sugar. I love it.

##### Responsibility

The importance of taking responsibility for oneself was highlighted. Those who had used various digital health services previously expressed that it helped them to take more action in their self-management. However, this was something they kept secret and did not always tell their diabetes nurse, since she might apprehend it as being critical of her advice. They also forced the importance of being seen as capable and responsible by the diabetes nurse, something that included that they accepted the consequences of even unhealthy choices. These participants had often got the advice from their diabetes nurses not to trust information on the internet and felt that using apps was in a gray zone, almost forbidden. Nevertheless, the participants described how it had helped them:

It [the app] helped me to take responsibility for a healthier behaviour; I believe I became more confident in myself since I started to use it. Much more than when I got my diabetes diagnosis.

#### Empowerment

A number of areas related to eHealth were found important for the management of the participants’ own health. They viewed applications and digital tools as powerful aids for understanding and becoming more aware, which enabled them to take control of their disease. Tracking their symptoms and treatments using diabetes apps and participation in online forum discussions provided them comfort. They learned of peers from online support groups by sharing what symptoms helped them take steps to adjust living with T2D, what types of treatment they used, and how this worked to strengthen them. As well-informed patients, they could more easily discuss and request different treatments with health care providers. The subcategories related to this category are *Knowledge*, *Participation*, *Engagement*, and *Freedom*.

##### Knowledge

Increased knowledge was highlighted as an important goal for managing T2D. The participants expressed that they preferred better collaboration between themselves and health care professionals. They saw themselves as knowledgeable, capable, and responsible for their own health and self-management. Now, knowledge enabled them to make informed choices, which could lead to better control, something the use of apps could facilitate. Gaining knowledge at one’s own pace was seen as a benefit.

I can get the knowledge I want about type 2 diabetes [on the internet], and make up my own goals, step by step at my own pace [using an app]...without having to discuss everything with the diabetes nurse.

##### Participation

Digital health services were perceived as providing opportunities for increased participation, since they could discuss their condition with people other than health care professionals. Some gave examples of their adult children’s increased participation when they lived far away. Using a mobile app that supported management of diabetes, the adult children could be updated online and follow the illness process at a distance. They could also easily get in touch with people with diabetes who they could contact through various Web-based portals for patients:

I especially enjoy being able to reason with others with the same problems on different patient forums. It is a kind of social networking, though I do not leave home often...

##### Engagement

Digital health services and devices made the participants more engaged through an increased awareness about the disease and needs for improved self-management. It was described that they traditionally met a doctor and a nurse semiannually. Between those visits, the disease-related information was easy to “forget,” and thereby they did not focus on changing habits. Due to an increased use of digital devices, they viewed personal visits at the health care center as unnecessary:

I feel more engaged now [using an app for self-monitoring]...I don´t always have to visit the primary health centre if I have problems, some things can be solved through eService on the primary healthcare centres website...

##### Freedom

Using digital health services was expressed as increasing the participants’ freedom. They gave examples of the freedom that was related to 24-hour service online. They did not have to wait until the next morning or a Monday, when the diabetes nurse was available if they had problems or had questions during the weekend:

Anytime during all hours I have the freedom to reflect and get feedback [from patient forums] on my thoughts. I do not have to wait until the next day when the primary healthcare centre opens as I did before.

#### Security

Digital health service was experienced as offering security. Safeguard components as passwords, encryption systems such as an e-ID (BankID or Mobile BankID), and similar technical safeguards for authorization or access controls strengthened the view of technology as something positive that protected the participants. The subcategories related to the category *Security* are *Confidentiality* and *Privacy*.

##### Confidentiality

The participants expressed worries and concerns about the following: that people from their community could witness them visiting the primary health care center and this could endanger their confidentiality. It could have personal consequences if information about them, known by neighbors, could get leaked to health care professionals, for example, about their families and social circumstances not known by a health care professional. In the next step, this information could get leaked to employers or maybe insurance companies. Sometimes they withheld information from health care professionals because of confidentiality concerns and also could avoid personal visits to the health care center. Web-based health care services were described as more secure, with personal log-ins, which was seen as trustworthy, and were at times perceived as better than the traditional face-to-face visits:

I trust that all information about me is kept confidential, even if it is online...but I do not know if I can trust that only authorised persons at the healthcare centre have access to my medical records...I mean, my neighbour works there as a secretary...

##### Privacy

It was highlighted that when digitized health is discussed in the media or in popular scientific literature, the ethics, security, and privacy risks are often questioned. Despite this, the participants were not worried. Instead, they expressed that lack of privacy was a barrier to visiting health care centers in small communities. Participants mentioned breaches of their privacy and had experienced that fellow patients took mobile photos in the waiting room and put them on Facebook. Using Web-based health services, they did not have to “advertise” their problems to other patients in the waiting room, and thereby, they did not feel as vulnerable and exposed:

When I sit in the waiting room, I could find it problematic to meet neighbours and others. I don’t want to expose myself as an ill person to them...I think I would prefer online meetings with my nurse.

### Concerns

Within the domain *Concerns*, which referred to the more negative side of the participants’ perceptions of using digital health services for self-management support, the categories *Ambivalence* and *Uncertainty* were highlighted.

#### Ambivalence

The participants expressed ambivalence concerning using digital health services and digital devices such as apps or iSMS. Mostly, it concerned feelings of lacking confidence and not being able to manage the technology. Furthermore, they had too little training, wanted support, and therefore avoided digital devices if they could. The subcategories related to the category *Ambivalence* are *Insufficient Support* and *Lack of Digital Skills*.

##### Insufficient Support

Being afraid of the new technologies as well as having limited or insufficient technological support increased the risk of not getting the medical advice participants needed. They therefore preferred face-to-face meetings with health care professionals. They did not have any family members or friends who could support them, and therefore, they were afraid of having technical problems.

What if something goes wrong?

##### Lack of Digital Skills

Participants expressed an ambivalence and reluctance toward using digital technology. The reason was expressed as having a lack of digital competence and skills. They also mentioned poor technological design as a barrier to navigate websites and apps. Participants stated that they had difficulties using their smartphones due to physical problems such as sight loss or tremor.

It´s too difficult to use for me, I can´t even type [on the smartphone].

#### Uncertainty

Digital systems in general were questioned by participants. They felt uncertain whether they could trust information they came across on the internet, and they were afraid of problems with eHealth services due to unreliable internet connections. The subcategories related to the category *Uncertainty* are *Distrust of Information* and *Unreliability*.

##### Distrust of Information

Participants saw no value in using technology to manage their health. Furthermore, they did not always trust the quality and authenticity of the information on websites they found and whether these websites provided accurate and detailed information about diabetes management. It was considered unsafe to rely entirely on the Web-based information that was available since the content could be medically incorrect and potentially endanger their health.

I mean, how can I be 100% sure that the information online is correct? It could be fatal.

##### Unreliability

Participants highlighted the unreliable and unstable connections, both on wired or wireless broadband with an internet turning on and off rapidly and slow when working. They also said that the lack of internet access through wired or wireless broadband technologies in their homes made it impossible to rely on and use the computer or smartphone for eHealth purposes. Participants expressed that even the primary health care service could not guarantee reliable computer systems:

What if there’s a system failure due to a crash or virus, and there will be loss of data? Or an unstable connection? Can the system be really secure?

## Discussion

### Principal Findings

This study has provided insight about the perceptions that people with T2D may have about using ICT and digital health services for self-management support, and the findings show that the participants are mainly positive, but they have mixed feelings regarding use of eHealth services and digital devices irrespective of whether it concerned a Web or mobile app. On one hand, they experienced potentials such as increased involvement, empowerment, and security; on the other hand, they expressed concerns such as ambivalence and uncertainty. One explanation for the variation in perceptions of using digital health services or eHealth services for self-management support could be the participants’ differing capabilities such as education and computer training and experience. From literature we know that age, gender, as well as SOS situations influence people’s perceptions [32-34].

Several studies report that eHealth is promising with regard to self-management support and that people with chronic conditions desire tools that effectively reduce the limitations of life caused by disease [41-43]. Alpay et al [44] concluded that by removing barriers of time and geographical distance in health care services—using digital and technological services such as video consultations and telehealth—the patients gain flexibility. They get an easier and more convenient access to health care, they may even have fewer time-demanding health care center visits, and finally, patients can receive care at a location that does not require transportation and in an environment that can be experienced as less threatening.

Regarding the category *Involvement*, our results highlight that self-monitoring may increase patients’ independence. Similar results are reported by Holtz and Lauckner [45], and by Alvarado et al [46], who showed that people with diabetes could adapt easier to their condition by using their mobile phones in self-monitoring and management of diabetes. Kruis et al [47] presented that innovative eHealth self-management solutions can support or improve independence among people with chronic conditions. Ahern et al [48] concluded that the potential of patient technologies can only be accomplished by motivating patients to become more engaged and responsible for their own care. In a study by Nijland et al [42], the authors argued that interactive eHealth applications must be continuously changed and developed to promote individual self-care, through feedback and exchange of information, something that is in line with the value of independence. Interactive eHealth tools designed to provide feedback on patients’ self-monitoring appear to engage patients the most, since personalized and interactive features stimulate active participation by both patients and nurses. Nijland et al [42] reported that the diabetes patients in their study felt better monitored by the feedback they received and were therefore more motivated to take a more active role in the self-management of their illness—something that also led to increased independence.

Regarding the category *Empowerment*, our results suggest that use of interactive eHealth platforms seems to have a potential to increase patient empowerment through increased knowledge, participation, engagement, and freedom. Our findings support previous studies that report that empowerment can be improved using digitized approaches in health care [5,44,49]. Empowerment implies participation and responsibility through increased awareness and knowledge [50,51]. Self-efficacy is an important aspect of empowerment and relates to change in behavior, which is important for self-management in chronic conditions [52]. Patient empowerment and PCC are closely related complementary concepts. These do not oppose each other, and indeed patient empowerment can be achieved through PCC [53]. Both patient empowerment and PCC are emphasized by health researchers and policy makers and expressed in care policy documents nationally and internationally [7,20]. Furthermore, it has been suggested that PCC increases patient outcomes and satisfaction in chronic illnesses [54,55] and T2D [54]. Thus, using the Web for medical and health facts is an approach in health care that can support empowerment and is facilitated by a shift to PCC that can subsequently improve self-management [25,30,55,56]. Digitized access increases patient empowerment and enables them to participate more actively in making better informed choices regarding their health in interaction with health care. Technological advances for self-monitoring are changing the conditions for chronic disease management. The use of different communication tools and interactive platforms may improve patient participation in decision making and facilitate for patients to communicate easily with health care professionals [49,57]. Medical and health information on the internet, digital health that patients use as in-home monitoring, virtual consultations, and mobile apps are also available to users 24 hours a day, 7 days a week [58] to provide alternatives to them apart from the primary health care centers, and this gives a certain degree of freedom [59]. However, a benefit for health care professionals using digitalized technology in self-management support is the option to be in contact with patients more frequently than semiannually or annually, as is common today [60,61].

Regarding the category *Security*, our results shows that participants in this study experienced that use of Web-based technology was seen as something safe and reduced privacy exposures, which is confirmed by other studies [62,63]. Participants were not bothered much about security concerns; they trusted that the different technical safeguards, such as passwords or encryption systems, were safe enough. Similar results are reported by Spanakis et al [63] who stated that most patients seem to be willing to disclose information relevant to their condition to their health provider, with no particular awareness of how the patient information is transferred. The use of digital health services can also reduce the number of visits to the health care centers, something that can be experienced as stressful, time-consuming, and expensive. Fewer face-to-face visits might also imply changes in the patients’ perception of self-management support as well as reconfiguring work activities for the diabetes nurse [64]. Encouraging patients to share their self-monitored data with the diabetes nurse to a higher degree may become a trade-off for fewer visits, thus having health economic implications. This is in line with a study by Eland-de Kok et al [65] who showed that adapted and person-centered support increased more than semiannual visits. This may lead to quality improvements and a higher priority for those patients who need face-to-face visits the most. A literature review by Hardiker and Grant [66] showed that the use of different Web-based services depended on a number of factors such as the characteristics of users, the kinds of technological issues, characteristics of the digital health services social aspects of users, and the digitized services in use. This requires health care professionals to concentrate their efforts where they are needed most, by tailoring services to meet the needs of a broad range of users.

Regarding the category *Ambivalence*, our results highlight that some of the participants stressed concerns regarding, for example, lacking digital skills and knowledge about how to use digital health services, which is in line with other studies [67,68] that have also reported an existing age-related digital division. This division concerns everything from the design of the digital device and screen design to complex commands and procedures, including inadequate training and instructions that can prevent older people from interacting with digital systems. Czaja and Lee [67] reported that predictors of not using digitized technology were primarily the very old with cognitive decline associated with different aging processes such as vision impairment, and attitudes such as anxiety about computer use and the perception that technology is not useful to them, both of which are compatible with our results. Usually participants in our study were also reluctant about using digital health services and preferred face-to-face meetings with health care professionals. Similar results are reported by Currie et al [27] who conclude that digitized solutions are not the key for every patient and thus do not have the same impact as a face-to-face meeting with health care professionals, since they may create feelings of loss of proximity for some patients. The lack of proximity in digital health services is also highlighted in other studies and is a challenge to overcome. Video consultations could sometimes compensate for the lack of proximity in digitized meetings [69,70]. Technological barriers could therefore be solved and personalized to meet the needs of those who have physical barriers such as cognitive, sensory, and motor deficits.

Regarding the category *Uncertainty*, our results highlighted that participants were ambivalent about their views of the reliability and quality of Web-based digital health information. Similar findings report individuals having difficulties using the internet to find complete and proper information concerning health issues. Not relying on Web-based information in making decisions about treatment and self-management, including whether or not to seek care, may negatively influence the user’s decisions [71,72]. In Sweden, 93% of the population have access to the internet at home, and outside the home, 71% connect to the internet using mobile phones or smartphones. Although access to internet is high in Sweden among the people aged 16 to 85 years, still 7% of households in Sweden do not have access to the internet. Those who have never used the internet are found mostly in the age group 75 to 85 years [73]. Even if Sweden is a country with very high internet access, we have interpreted limited access to internet connections or broadband as a factor that affects the usefulness of digital health services. This is concurrent with Currie et al [27] who reported problems for patients living in rural areas compared with those living in urban areas concerning the use of technology for health purposes. They highlighted challenges related to slow and unreliable broadband services. Fuji et al [74], on the other hand, conclude that instead of primarily focusing on issues concerning internet infrastructure or a lack of internet access in rural areas, focus should be placed on overcoming other concerns and barriers among the users.

Our results could guide such development. The result also indicates that future digital health solutions preferably should have high demands on functionality, personalization, and an easy-to-use design to be user-friendly. Self-monitoring and measurements should also be smooth to integrate with the health care records and communication channels. Furthermore, a “universal” digital solution does not exist. One size rarely suits everyone. To improve user customization, people with T2D from various socioeconomic backgrounds, gender, and ages need to be involved in the development of future digital tools.

### Strengths and Limitations

The findings in this qualitative study cast some light on the experiences of using various digital health services in self-management support among people with T2D treated in Swedish primary health care. We view our results as transferable to other groups of patients with similar lifestyle-related chronic conditions in societies similar to Sweden. However, according to Graneheim and Lundman [36], it is up to the reader’s judgment as whether or not the reported findings are transferable to other contexts.

We recruited 11 people with T2D for individual interviews, using a combination of purposive and subsequent sampling [75], which made it possible to expand the group of participants. However, there is a risk of bias, since our sample may consist of participants with an interest in eHealth. Despite that, our result pointed to a variation of perceptions about the use of eHealth services and could thereby be useful.

The majority of the participants were men, and the age range was 50 to 74 years. It is possible that the outcome of this study would have been different if more women had been included and if the age range had been different, including, for example, very old patients. Nevertheless, the participants in this study are representative of people with T2D and provided rich data.

There are no rules for how large the selection of participants should be in qualitative research methodology, but the selection is generally determined by the need for information data. In this case, it was considered that it had come to the stage where further data collection would not provide more knowledge and that the collected data was sufficient for the study. The saturation point was judged as reached. The term saturation derives from grounded theory, but it is also used in other qualitative approaches [76].

The interviews were conducted by the first author alone. However, all authors listened to and discussed the interviews and then were involved in interpretations at every step of the analytical process, something we believe has strengthened the trustworthiness of the study and resulted in a consolidation of the findings.

### Conclusions

The results from this study indicate that persons with T2D have diverse perceptions on using digital health technologies and eHealth services for self-management support. They are interested in digital health technologies and services for self-management support, however, ambivalence was also expressed. Our findings indicate that targeted training and support is needed to overcome barriers and that utilized devices for good reason should be personalized or carefully adapted to the specific situations at hand.

The use of digital health technologies for person-centered self-management support is challenging but can—if implemented appropriately—lead to increasing patient responsibility for their own health and strengthen patients’ empowerment and self-management capabilities. Although digital health technologies of today allow for innovative approaches, there are also ethical aspects to consider when new digital health tools or solutions and eHealth services are introduced in health care. Some people may neither wish to nor be able to use digital technology for various reasons on their own, whereas others see it as an important complement to or even substitute for the traditional health care visits.

## Abbreviations

eHealth: electronic health
ICT: information and communication technology
iSMS: interactive self-management support
PCC: person-centered care
SOS: socioeconomic status
T2D: type 2 diabetes
